# Intra-Articular Osteoid Osteoma of the Trapezoid Bone: A Rare Presentation Mimicking Wrist Synovitis

**DOI:** 10.1155/carm/7648066

**Published:** 2025-04-28

**Authors:** Khadija Baccouche, Rym Fakhfekh, Dhouha Khalifa, Haifa Hachfi, Cyrine Daldoul, Nejla Elamri, Elyes Bouajina

**Affiliations:** Rheumatology Department of Farhat Hached University Hospital, Faculty of Medicine of Sousse, University of Sousse, Sousse, Tunisia

## Abstract

Osteoid osteomas predominantly occur in the cortices of long bones, with the femur and tibia being the most commonly affected sites. However, they can occasionally present in atypical locations, such as the carpus, which can lead to diagnostic confusion with other conditions. This case report details an intraarticular osteoid osteoma in the trapezoid bone. Initial evaluations, including standard radiographs, joint ultrasound, and wrist MRI performed twice, initially pointed toward a diagnosis of wrist synovitis. This case underscores the diagnostic challenges posed by atypical presentations of osteoid osteomas. Given the edema present in the carpal bones alongside the synovitis, we performed a hand CT scan, which raised doubts about the appearance of the nidus and histopathological examination confirmed the diagnosis. Clinical symptoms, including pain and functional limitations, were completely resolved following surgical excision.

## 1. Introduction

Osteoid osteoma (OO) is a benign bone tumor distinguished by the production of unmineralized bone known as osteoid, constituting approximately 10% of all benign bone tumors. The tumor is characterized by a highly vascular central nidus made up of osteoid and woven bone, encased in a surrounding zone of reactive bone sclerosis. It predominantly occurs in the cortices of long bones, with the femur and tibia being the most commonly affected sites. However, OO can occasionally present in atypical locations, such as the carpus, which may lead to diagnostic confusion with other conditions. A review of the literature reveals only eight reported cases of OO located in the trapezium [1–3]. We report a case of diagnostic challenge involving an atypical, intra-articular OO in the trapezoid bone. This presentation mimicked wrist synovitis, leading to a delayed diagnosis.

## 2. Case

A 24-year-old right-handed female presented with a 3-year history of progressively worsening pain localized to the radial aspect of her left wrist. She reported no prior trauma to the wrist. The pain, which had gradually intensified, markedly impaired her daily activities and disrupted her sleep. Clinical examination revealed swelling of the wrist, tenderness on palpation, and a restricted range of motion, with no evidence of fever or systemic inflammation.

**Figure 1:**
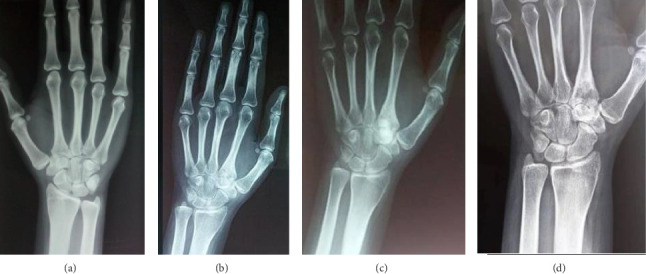
The Evolution of wrist radiography on the time. (a) Normal initial radiography, (b) joint space narrowing of the trapezoid and the second metacarpal bones, (c) well limited sclerotic lesions in the trapezoid and the base of the second metacarpal bones, (d) altering bone at the trapezoid and the second metacarpal bone.

**Figure 2:**
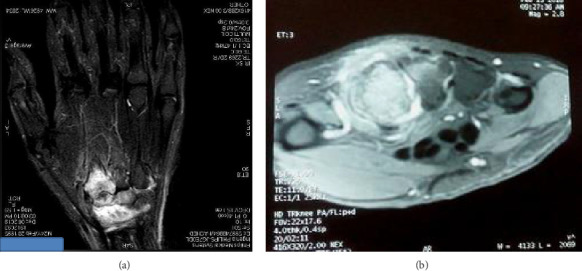
Magnetic resonance imaging (MRI) showing bone marrow oedema in the trapezoid and the second metacarpal bones, synovial effusion, and tissue oedema around the tumor: (a) coronal T2-weighted fat-saturated MRI sequences and (b) transverse T1-weighted gadolinium-enhanced MRI sequences.

Initial wrist radiography was normal (Figure 1(a)). However, after 20 months, repeat radiography revealed joint space narrowing in the trapezoid and the second metacarpal bone, without evidence of erosion (Figure 1(b)). This imaging finding suggested the possibility of either synovitis or erosive arthritis. Wrist ultrasound revealed grade 2 synovitis in B-mode and increased vascularity in Doppler mode. Magnetic resonance imaging (MRI), performed twice over the course of a year, consistently showed synovial effusion, bone marrow edema in the trapezium, trapezoid bone, and the base and proximal diaphysis of the second metacarpal bone, and tenosynovitis of the adjacent flexors and extensors (Figure 2). Additionally, bone scintigraphy demonstrated increased uptake of 99 mTc in the radial side of the left wrist. The patient was treated with a range of interventions, including oral anti-inflammatory drugs (Naproxen 550 mg/day), corticosteroids, multiple intra-articular steroid injections, and morphine up to 60 mg/day. Despite this comprehensive regimen, the patient continued to experience persistent, severe, and disruptive pain, with only partial relief provided by Naproxen.

Given the presentation of a highly painful wrist synovitis unresponsive to conventional treatment, and in light of negative results from immunological and tuberculosis investigations as well as the absence of fluid on joint aspiration, a synovial biopsy was undertaken. The histopathological analysis revealed nonspecific chronic inflammation.

**Figure 3:**
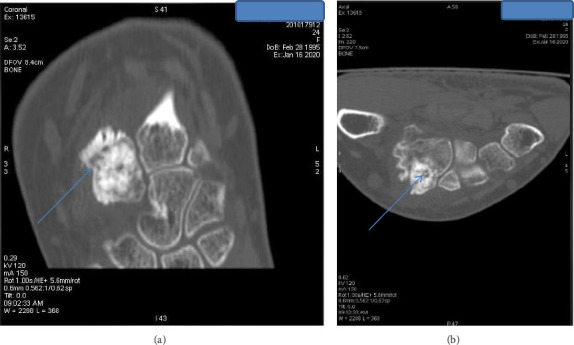
The coronal (a) and axial (b) computed tomography show an intra-articular nidus at the trapezoidal bone surrounded by sclerotic lesions at the trapezoidal and the base of the second metacarpal bones.

**Figure 4:**
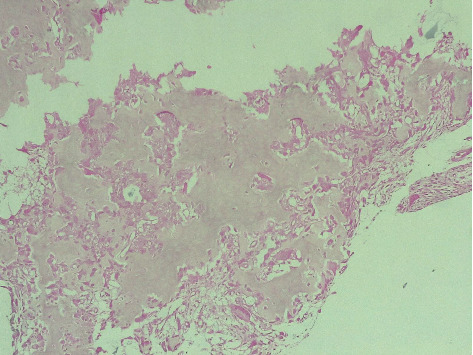
Histopathology of the osteoid osteoma (Haematoxylin and Eosin × 400).

Given the exclusion of early inflammatory rheumatic diseases (IRD) and joint infections, and the predominant unexplained bone edema observed on MRI, a computed tomography (CT) scan was warranted to further assess bone structure and investigate potential underlying lesions. The indication for CT was further reinforced by follow-up radiographs (Figure 1(c)), which revealed well-defined sclerotic lesions in the trapezoid and the base of the second metacarpal bones, raising suspicion of a benign bone tumor or another structural abnormality requiring further characterization. The CT scan (Figure 3) revealed an intra-articular focal lucent nidus in the trapezoid bone, surrounded by a sclerotic reactive lesion involving both the trapezoid and the base of the second metacarpal bones. This led to the diagnosis of an OO with atypical location and presentation. The patient underwent surgical treatment via a dorsal approach. Intraoperatively, it was observed that the OO in the trapezoid bone extended to the base of the second metacarpal bone. An en bloc tumor excision was performed without reconstructive bone surgery. The gross specimen revealed a red, granular area surrounded by sclerotic bone, measuring 1.5 cm. Histopathological examination identified a network of short, anastomosing trabeculae of osteoid, lined by regular osteoblasts with a few osteoclasts embedded in central hypervascularized loose connective tissue, confirming the diagnosis of OO (Figure 4). Following the surgical excision, the patient experienced immediate pain relief and a gradual improvement in wrist motion and function. Fourteen months post-surgery, she reported no discomfort or functional limitations in the affected extremity and had returned to work. Postoperative radiography showed changes in the bone structure of the trapezoid and the second metacarpal bone (Figure 1(d)).

## 3. Discussion

OO typically presents in the second or third decade of life, with a predominance in males [3]. While it most commonly affects the long bones, atypical locations have also been documented [1–4]. OO can be localized cortically, trabecularly, or subchondrally/intra-articularly [1, 2]. In our case, we describe an intra-articular OO of the trapezoid bone, mimicking an inflammatory wrist condition. Intra-articular OO of the trapezoid bone is exceedingly rare, with only eight cases reported in the literature [2–7], and has been recognized as a possible differential diagnosis for wrist arthralgia [3]. In 90% of carpal OO cases, diffuse wrist pain is observed [2, 4], often accompanied by swelling, typically attributed to tenosynovitis, and a reduced range of motion [1, 2, 4], as was evident in our patient. While imaging techniques like CT have high sensitivity for diagnosing OO, they can also create a misleading context, potentially delaying appropriate management due to the overlap with IRD symptoms and the atypical location, such as intra-articular OO [1, 3]. Intra-articular OO reported in the literature often present with normal initial radiographic findings [2, 3], as observed in our patient. Given the initial presentation that resembles arthritis, MRI is typically employed for a more comprehensive assessment. However, since bone marrow edema is the predominant MRI feature in cases of bone tumors, it can obscure the correct diagnosis and potentially lead to interpretive errors. Indeed, previous reports have noted misdiagnosis of OO due to the presence of bone marrow edema [10]. MRI typically reveals not only bone marrow edema but also synovial thickening and tenosynovitis, which are characteristic of intra-articular OO [2, 3]. These findings help differentiate intra-articular OO from extra-articular variants with high sensitivity and specificity [8]. Technetium-99m bone scintigraphy reveals a focal area of increased uptake in all three phases; however, this finding lacks specificity [3]. CT with thin slices is crucial for diagnosing carpal OOs, providing superior diagnostic detail and aiding in surgical planning compared to conventional radiography and MRI [3]. Our case highlights the diagnostic challenges associated with an OO exhibiting atypical localization (intra-articular in the wrist) and presentation (hyperalgesic and disabling arthritis). This difficulty arises because CT, which is crucial for diagnosing OO, is not typically the first-line examination for arthritis, and other imaging modalities often lack specificity. In summary, our results suggest that OO should be considered in the differential diagnosis of chronic joint pain or arthritis, particularly in cases where there is persistent and extensive peri-articular bone marrow edema, negative etiological investigations, and pain unresponsive to conventional treatments.

Many surgical techniques have been defined and are selected on a case-by-case basis [3–7]. Conventionally, OO is removed either by en bloc resection or curettage, with bone grafting sometimes necessary. In recent years, minimally invasive techniques such as CT-guided percutaneous core-drill excision, radiofrequency ablation, or laser photocoagulation have emerged as effective alternatives to conventional surgical methods [9, 10]. These treatments typically result in almost immediate pain relief; however, incomplete removal of the nidus may lead to symptom recurrence or persistence [2, 3].

Gravina et al. reported a case in which bone reconstruction was performed using bioactive glass following the excision of a larger OO in the trapezium [11]. Their approach aimed to fill the bone defect while minimizing donor site morbidity, particularly in a young patient. The bioactive glass served as an osteoconductive material, facilitating bone regeneration and ensuring structural stability. By contrast, in our case, reconstruction was not required due to the small size of the tumor and the limited bone defect left after curettage. This decision reflects the importance of tailoring the treatment approach to the specific characteristics of each case.

## 4. Conclusion

This case underscores the diagnostic challenges of intra-articular OO in atypical locations like the trapezoid bone. Initially misinterpreted as wrist synovitis, the diagnosis was delayed due to overlapping symptoms and inconclusive imaging. Advanced imaging, particularly CT, ultimately confirmed the diagnosis, and surgical excision resulted in complete symptom resolution. This highlights the need to consider OO in cases of chronic joint pain with persistent bone marrow edema, ensuring timely diagnosis and appropriate treatment to improve patient outcomes.

## Data Availability

The data that support the findings of this study are available from the corresponding author upon reasonable request.

## References

[1] Rolvien T., Krause M., Zustin J. (2019). Intra-Articular Osteoid Osteoma Accompanied by Extensive Bone Marrow Edema. A Clinical and Micro-Morphological Analysis. *Journal of Bone Oncology*.

[2] Koutas K., Papagiannis S., Giannatos V., Stavropoulos T., Kokkalis Z. (2023). Osteoid Osteoma of the Trapezium: A Rare Case Report and Literature Review. *Cureus*.

[3] Jafari D., Shariatzade H., Mazhar F. N., Abbasgholizadeh B., Dashtebozorgh A. (2013). Osteoid Osteoma of the Hand and Wrist: A Report of 25 Cases. *Medical Journal of the Islamic Republic of Iran*.

[4] Girard J., Becquet E., Limousin M., Chantelot C., Fontaine C. (2005). Ostéome Ostéoïde de l’os Trapézoïde: À Propos d’un Cas et Revue de la Littérature. *Chirurgie de la Main*.

[5] Tonogai I., Hamada Y., Yasui N. (2012). A Case of Osteoid Osteoma of the Trapezoid Bone: The Efficiency of Dynamic Magnetic Resonance Imaging for the Detection of Osteoid Osteoma Localized at the Atypical Site. *Hand Surgery*.

[6] Jafari D., Najd Mazhar F. (2012). Osteoid Osteoma of the Trapezoid Bone. *Archives of Iranian Medicine*.

[7] Tricoire J. L., Duport M., Puget J., Mazières B., Chiron P., Utheza G. (1991). Ostéome ostéoïde du trapézoïde. *Annales de Chirurgie de la Main et du Membre Superieur*.

[8] Germann T., Weber M.-A., Lehner B. (2020). Intraarticular Osteoid Osteoma: MRI Characteristics and Clinical Presentation Before and After Radiofrequency Ablation Compared to Extraarticular Osteoid Osteoma. *Röfo*.

[9] Aiba H., Hayashi K., Inatani H. (2014). Conservative Treatment for Patients With Osteoid Osteoma: A Case Series. *Anticancer Research*.

[10] Gökalp M. A., Gözen A., Ünsal S. Ş, Önder H., Güner S. (2016). An Alternative Surgical Method for Treatment of Osteoid Osteoma. *Medical Science Monitor*.

[11] Gravina P., De Francesco F., Pangrazi P. P., Gigante A., Riccio M. (2022). A Large Osteoid Osteoma of Trapezium: A Regenerative Approach and a Review of Literature. *Journal of Hand Surgery Global Online*.

